# Supplementary material for: Stoichiometry-Dependent Specificity in Biotin Enrichment: A Benchmarking Framework for Proximity-Labeling Proteomics
Source: Anal Chem. 2026 Sep 4;98(36):26636–48. doi: 10.1021/acs.analchem.6c03011 (PMC13584629; doi:10.1021/acs.analchem.6c03011)
Supplement: Supplementary file 1 [file ac6c03011_si_001.pdf]

Supporting Information

Stoichiometry-dependent specificity in biotin enrichment: a benchmarking framework for proximity labeling proteomics

Christopher Árpád Zala[a,b#], Maria Cristina Trueba Sanchez[a,b#], Jelle van den Bor[c], Tijmen Willemsens[a.b], Frederik Johannes Verweij[c], Maarten Altelaar[a,b], Kelly E. Stecker[a,b*]

[a]Biomolecular Mass Spectrometry and Proteomics, Utrecht Institute for Pharmaceutical Sciences, University of Utrecht, Padualaan 8, 3584CH Utrecht, The Netherlands.

[b]Netherlands Proteomics Center, Padualaan 8, 3584CH Utrecht, The Netherlands.

[c]Department of Cell Biology, Neurobiology and Biophysics, Utrecht University, Padualaan 8, 3584CH Utrecht,, The Netherlands.

[#]These authors contributed equally to this work.

[*] Corresponding author, email: k.e.stecker@uu.nl

Table of contents

Supplementary Figures

Figure S1. Effects of wash conditions and bead types on enrichment efficiency.

Figure S2. Change of human background proteins across different biotinylation percentages.

Figure S3. Biotinylated sender cells and EVs are specifically enriched for EV proteins.

Figure S4. Proteins enriched in receiver cells treated with biotinylated EVs correspond to high-ranking proteins in EV enrichment.

Figure S5. Decreased biotin content of UID-TSPAN3 EVs leads to no detectable enrichment of biotinylated proteins after feeding to receiver cells.

Figure S6. Proteins significantly upregulated are closely related.

Figure S7. DIA-NN settings used for searches.

Supplementary Table

Table S1. DIA window sizes used for DIA acquisition.

Table S2. Workflow recommendations for low-stoichiometry biotin enrichment optimizations.

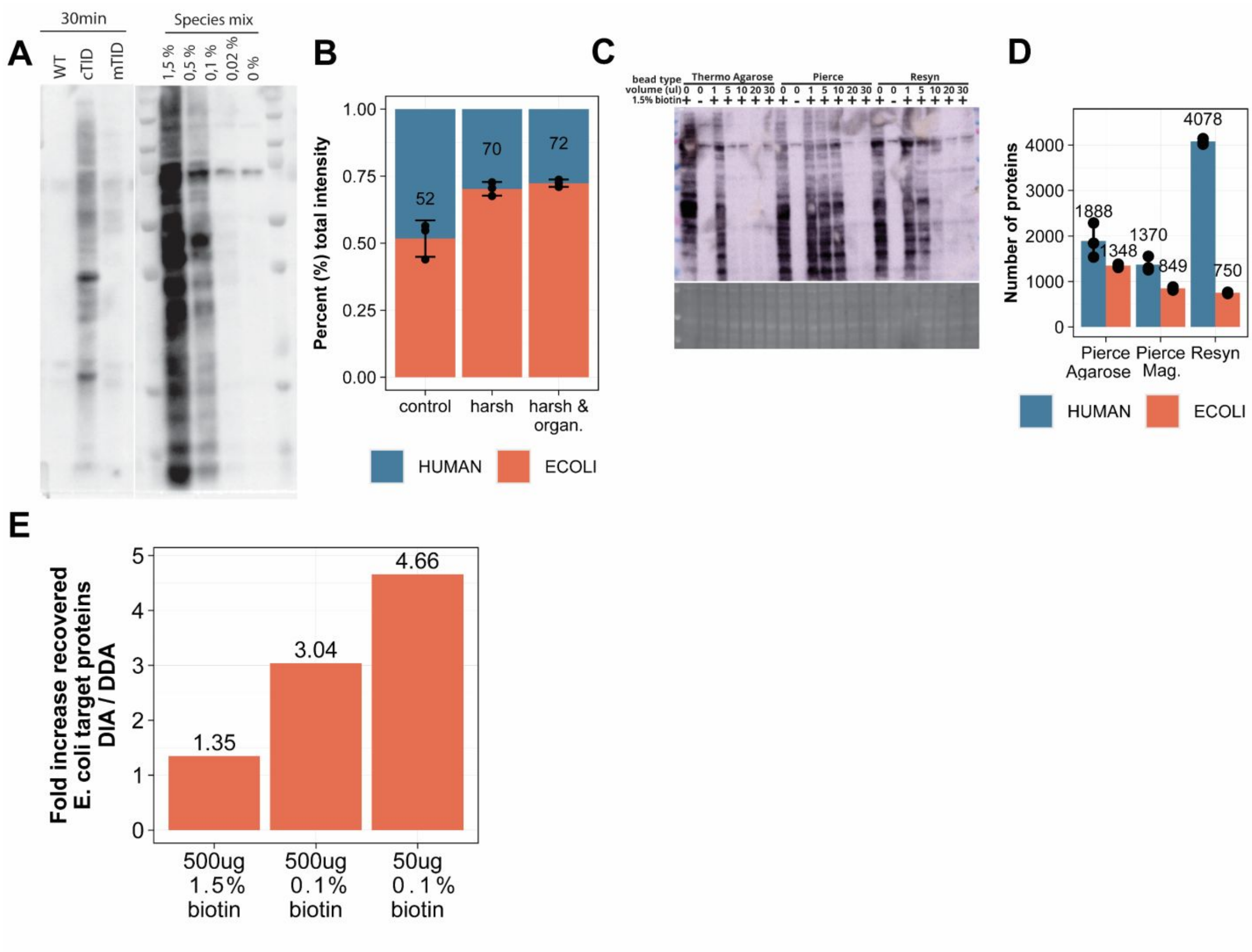

Figure S1. Effects of wash conditions, bead types and analysis method on enrichment efficiency and target recovery.

A) Western blot of cell lysates of cytosolic (cTID) or membrane-localized TurboID (mTID) samples after 30 min labeling, alongside biotinylated benchmark standards. Each lane contains 20 µg of the respective sample. Biotinylated proteins were detected with streptavidin–HRP. **B)** Stacked bar plots showing the percentage of total MS intensity attributed to *E. coli* (red, target) and human (blue, background) proteins after enrichment of 0.1% biotinylated *E. coli* protein lysate in 500 µg of human background with Pierce agarose beads across different wash conditions. Control wash contains low percentages of detergent, harsh wash contains high percentages of detergent, harsh & organic wash contains high percentages of detergent with subsequent organic solvent wash. Error bars represent the standard deviation; n = 3 samples. **C)** Top: Westernblot of 500ug of 1.5% biotinylated *E. coli* lysate in human background was depleted with different volumes of either Pierce agarose, Pierce magnetic or Resyn beads. 20ug of initial sample was loaded on the WB. Biotinylated proteins are detected using streptavidin-HRP. Bottom: Ponceau S staining shows total protein loading of the corresponding blot. **D)** Bar plot showing the number of *E. coli* (red, target) and human (blue, background) proteins detected by LC–MS after enrichment of 500 µg of 1.5% biotinylated *E. coli* lysate in human background using different bead types. **E)** Bar plots showing the fold increase in *E. coli* target protein recovery obtained by DIA compared to DDA for selected samples with

different biotinylation percentages and protein input amounts. Error bars in panels **B** & **D** represent the standard deviation; n = 3 samples.

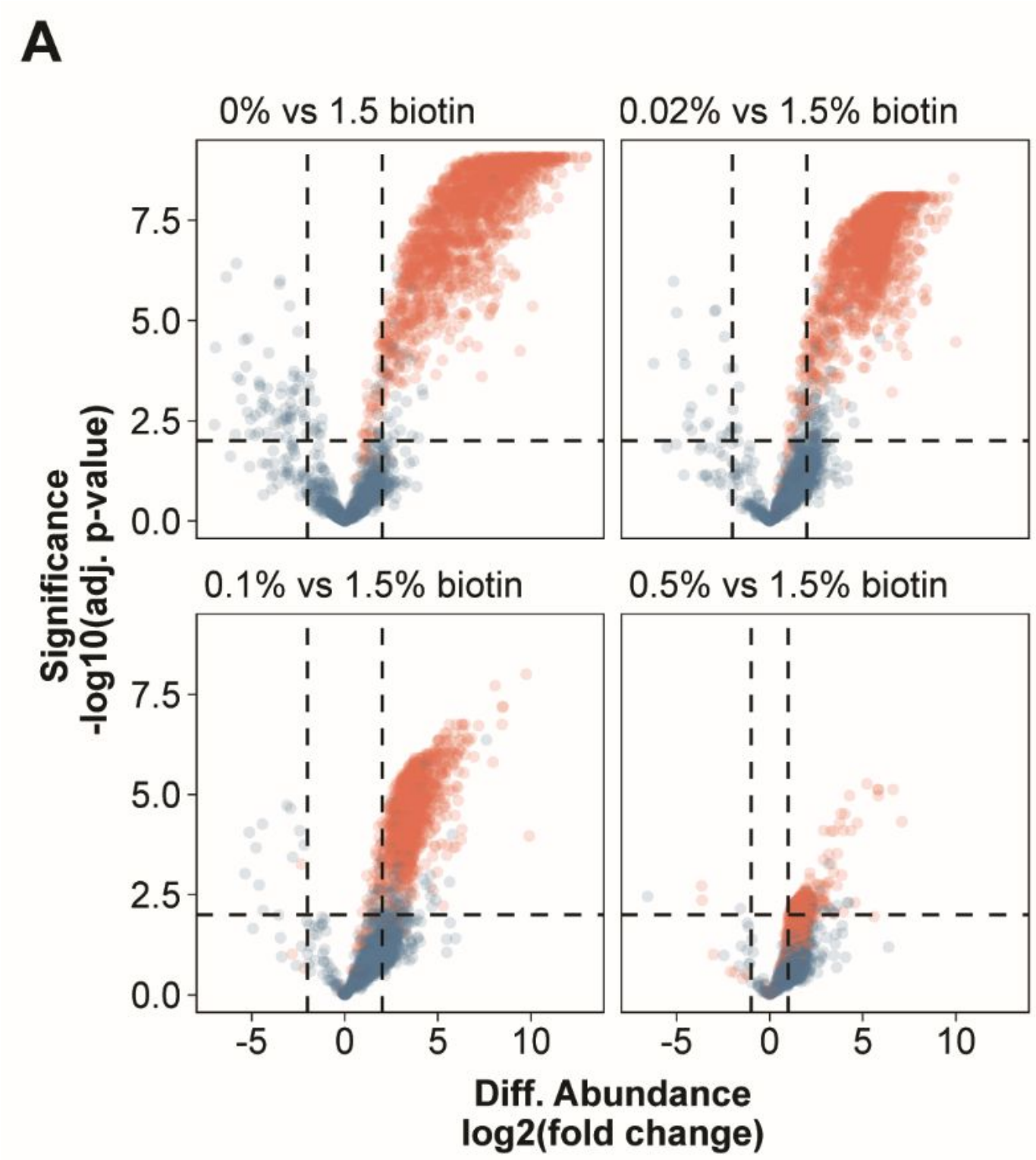

Figure S2. Change of human background proteins across different biotinylation percentages.

A) 500ug of input sample at different biotinylation percentages was enriched with Pierce agarose beads (data shown previously shown in Figure 1 B and E). Volcano plots showing differential protein abundance versus –log10(adjusted p-value) for pairwise comparisons of biotinylation percentages of *E. coli* (red, target) and human (blue, background) proteins. P-values were calculated using a moderated t-test from the limma package, and adjustment for multiple testing was performed using the Benjamini-Hochberg method. Horizontal lines indicate an adjusted p-value threshold of 0.01. Vertical lines indicate log2 fold change thresholds: 2 for comparisons of 0, 0.02, and 0.1%, and 1 for the 0.5% comparison.

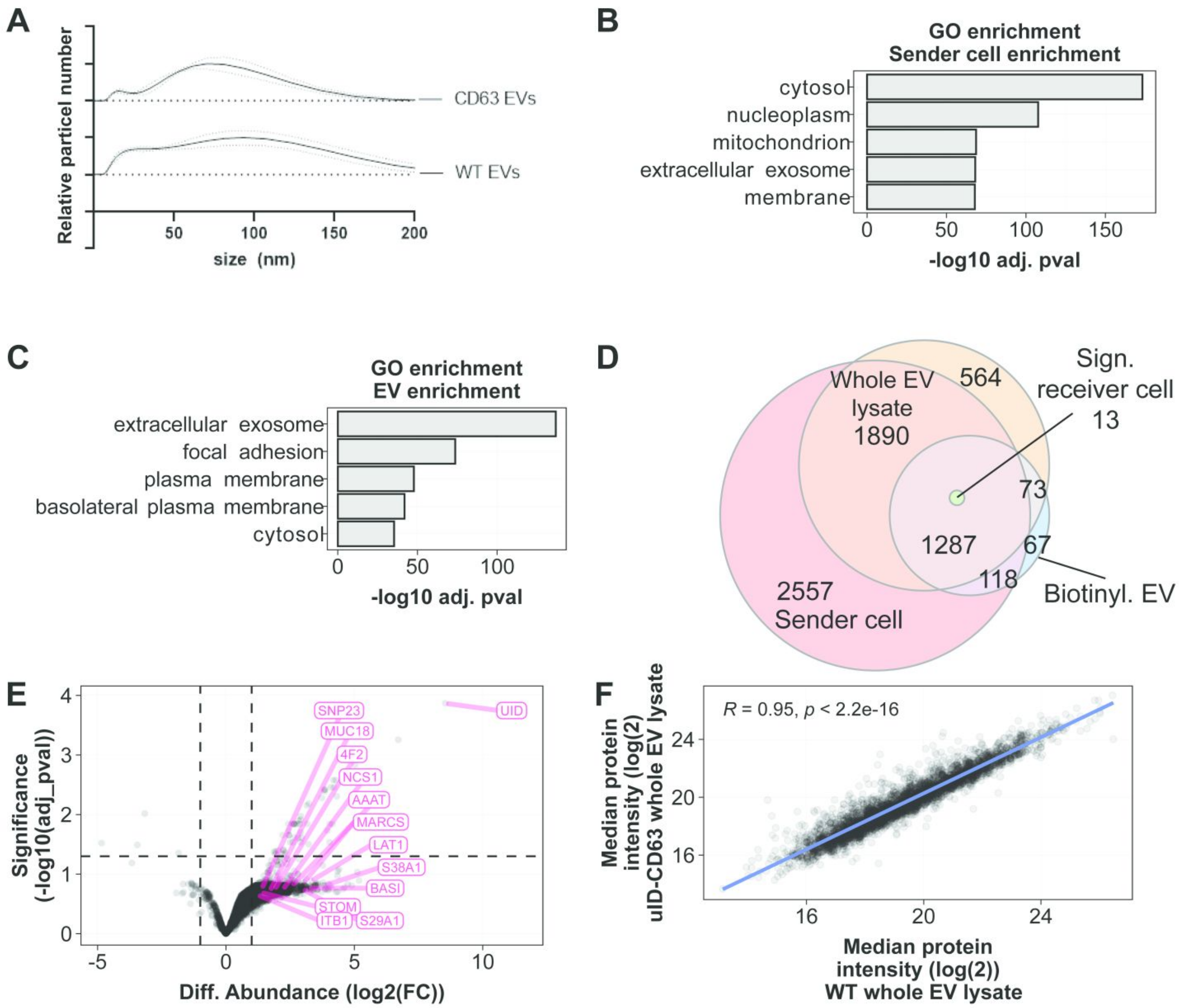

Figure S3. Biotinylated sender cells and EVs are specifically enriched for EV proteins.

A) Particle size distribution profiles of CD63 EVs and wild-type (WT) EVs measured by dynamic light scattering. The x-axis shows particle diameter (nm) and the y-axis shows relative particle number (arbitrary units). **B&C)** Gene Ontology (GO) enrichment analysis of proteins significantly upregulated in uID-CD63 compared to wild-type (WT) samples (see volcano plots in Figure 4C). Significantly upregulated proteins were tested against the whole human proteome as background. Enrichment was assessed using Fisher's exact test. Enriched GO terms in biotin-enriched sender cells (B) and in biotin-enriched EVs (C). **D)**Venn diagram showing overlap of proteins detected in sender cell enrichment (red circle), biotinylated proteins detected in EVs (light blue circle), biotinylated proteins found to be upregulated in receiver cells after feeding with biotinylated EVs (green circle) and proteins found in whole EV lysates (orange circle). **E)** Volcano plot showing differential protein abundance versus significance (–log10(adjusted p-value)) of whole EV lysate (D). Comparisons are always CD63-UID versus WT. Proteins significantly upregulated in receiver cell enrichment are highlighted in pink. **F)** Scatter plot showing the correlation of median protein intensities between wild-type (WT; x-axis) and uID-CD63 (y-axis) samples derived from whole EV

lysates. Each point represents a quantified protein. The Pearson correlation coefficient is displayed at the top of the plot, indicating the strength of the linear relationship between the two conditions.

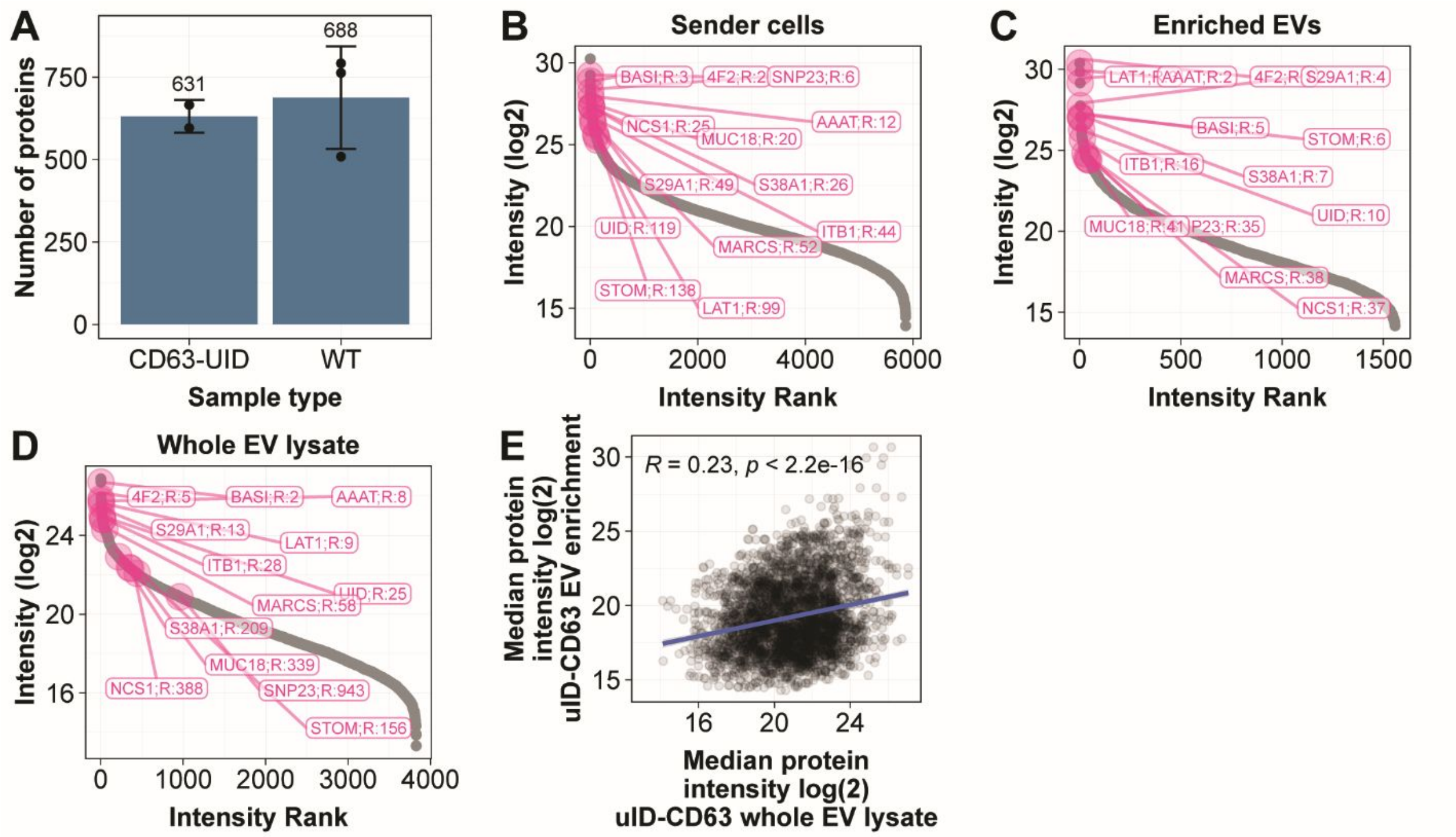

Figure S4. Proteins enriched in receiver cells treated with biotinylated EVs correspond to high-ranking proteins in EV enrichment.

A) Number of proteins retrieved after biotin enrichment of receiver cells fed with EVs biotinylated by CD63-UID construct or with WT EVs. Error bars represent the standard deviation; n = 3 samples. **B-D)** Rank plots of sender cells and EVs, showing log2 protein intensity (y-axis) versus intensity rank (x-axis). Panels B&C: biotin-enriched samples of sender cell lysates (B) and isolated EVs (C); panel D: whole EV lysates. Proteins significantly upregulated in receiver cells after biotinylated EV treatment are highlighted in pink. **E)** Scatter plot showing the correlation of median protein intensities between samples derived from uID-CD63 whole EV lysates (x-axis) and samples derived from biotin enriched uID-CD63 EV (y-axis). Each point represents a quantified protein. The Pearson correlation coefficient is displayed at the top of the plot, indicating the strength of the linear relationship between the two conditions.

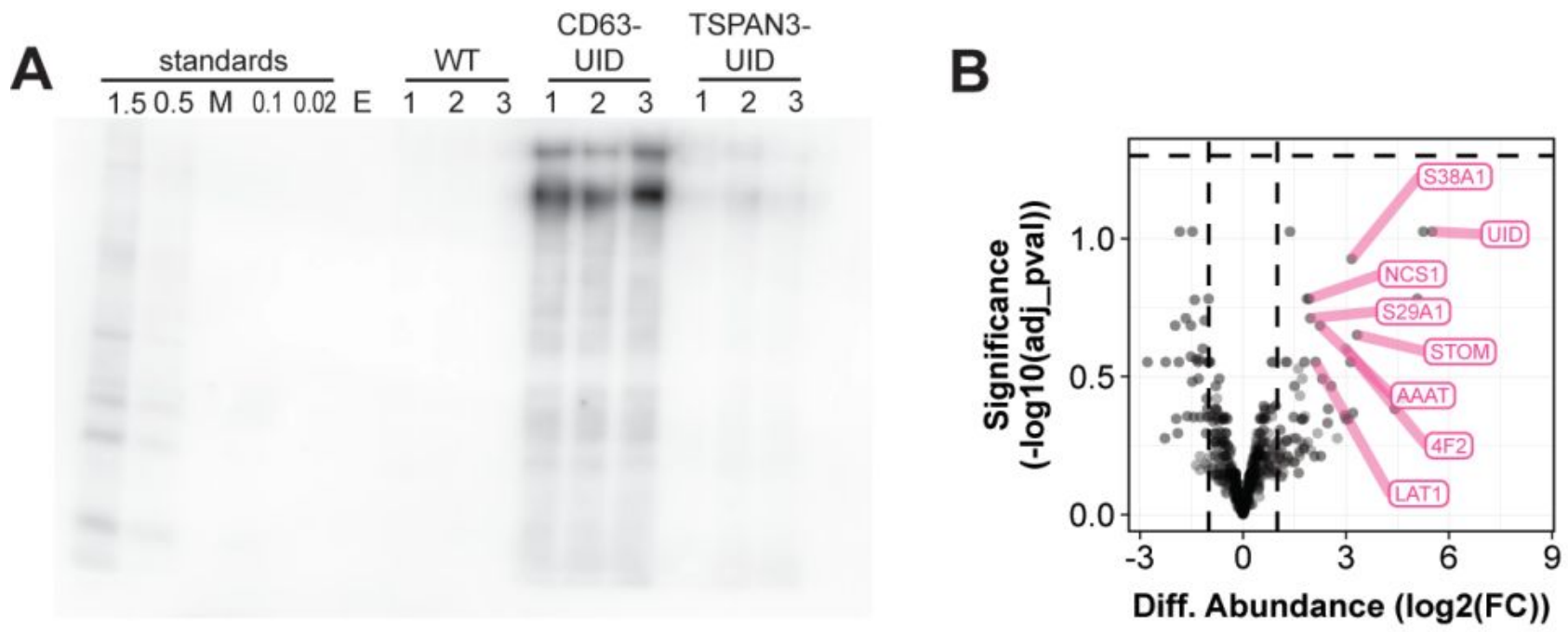

Figure S5. Decreased biotin content of UID-TSPAN3 EVs leads to no detectable enrichment of biotinylated proteins after feeding to receiver cells.

A) Western blot of isolated EVs alongside biotinylated benchmark standards. Each lane contains 20 µg of the respective sample. Biotinylated proteins were detected with streptavidin–HRP. WT referres to EVs isolated from WT HeLa cells, CD63-UID refers to EVs isolated from HeLa cells genetically modified with CD63-UID construct, TSPAN3-UID refers to EVs isolated from HeLa cells genetically modified with TSPAN3-UID constructs. Letter E marks empty lanes. **B)** Volcano plot showing differential protein abundance versus significance (–log10(adjusted p-value)) of receiver cells fed with TSPAN3-UID containing EVs. Comparisons is TSPAN3-UID versus WT. Proteins significantly upregulated in receiver cell enrichment fed with CD63-UID containing EVs are are highlighted in pink.

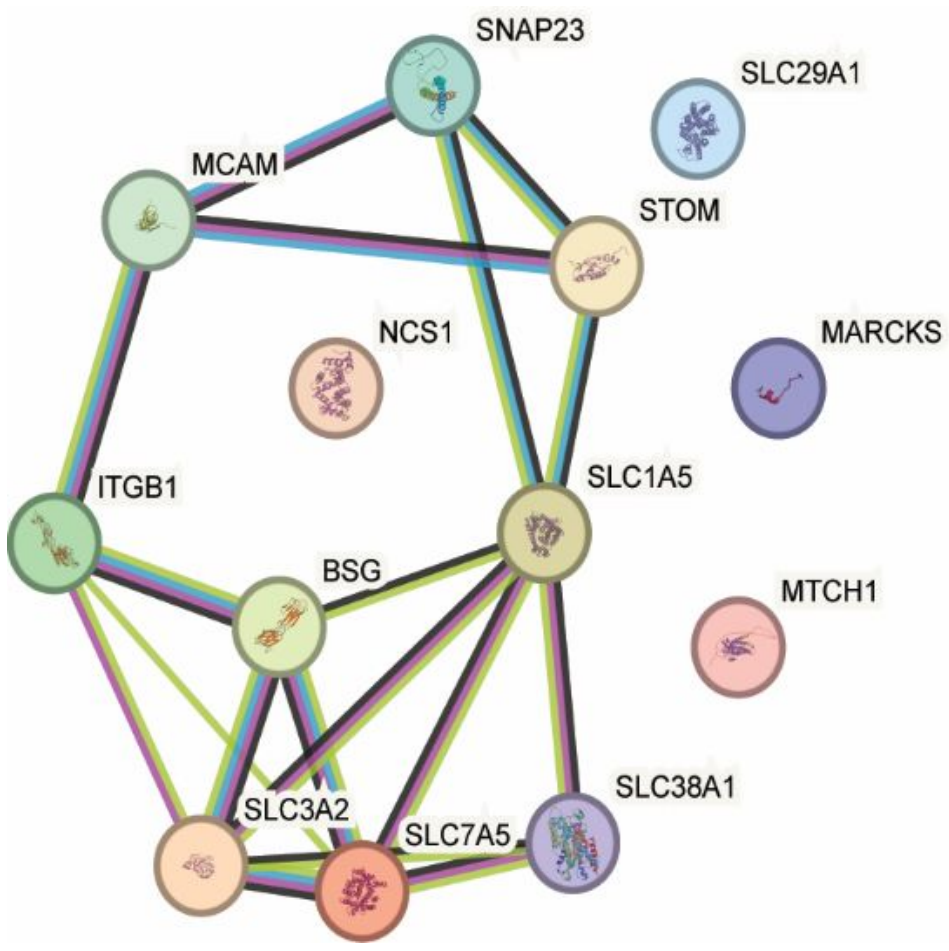

Figure S6. Proteins significantly upregulated are closely related.

STRING network analysis of proteins significantly upregulated in receiver cells following biotinylated EV treatment. Only interactions with a confidence score ≥ 0.4 are shown.

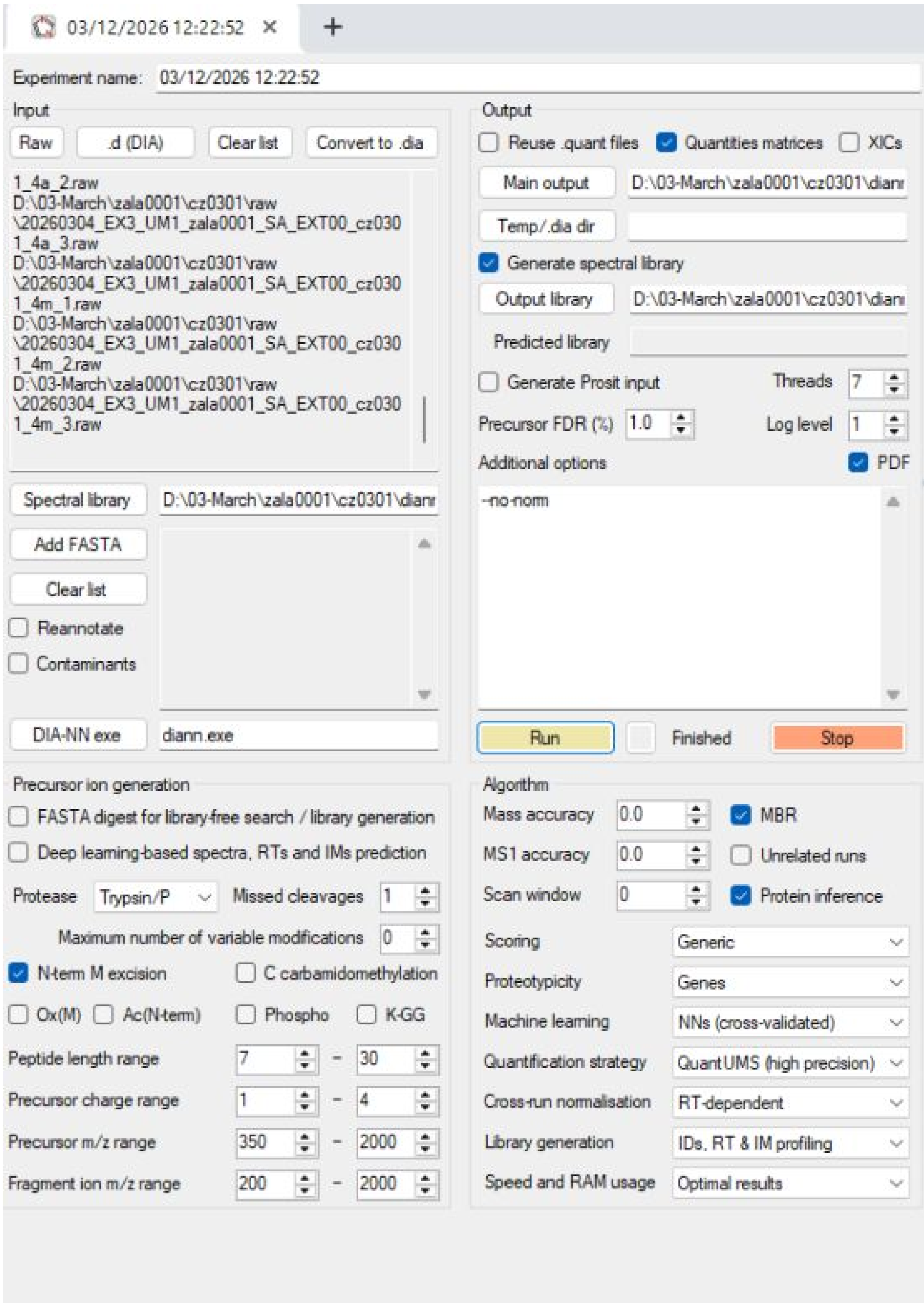

Figure S7. DIA-NN settings used for searches.

Table S1. DIA window sizes used for DIA acquisition.

m/z range
399-407
406-414
413-420
419-426
425-432
431-438
437-444
443-450
449-455
454-461
460-467
466-472
471-479
478-484
483-490
489-496
495-502
501-509
508-516
515-522
521-529
528-536
535-543
542-550
549-558
557-565
564-574
573-583
582-592
591-602
601-613
612-624
623-637
636-649
648-663
662-677
676-694
693-712
711-732
731-756
755-782
781-813
812-851
850-906
905-1000
999-1650

Table S2. Workflow recommendations for low-stoichiometry biotin enrichment optimizations.

Workflow stage	Recommendation
**Increase sample biotin stoichiometry**	Increase enzymatic labeling time.
	Increase labeling enzyme expression.
	Increase chemical labeling time.
	Increase labeling reagent concentration.
**Optimize biotin enrichment procedure**	Optimize the protein-to-bead ratio for each sample type.
	Verify complete depletion of biotinylated proteins WB using increasing bead amounts.
	Use the minimum bead amount required.
	Use wash buffers containing detergent and organic solvent to reduce nonspecific binding.
	Evaluate Pierce Neutravidin agarose beads alongside the affinity matrix routinely used in your laboratory.
**Optimize MS measurement and data analysis**	Inject the maximum feasible sample amount, even if bead-derived peptides dominate the MS signal.
	If bead-derived peptide overloading limits LC-MS performance, consider manufacturing protease-resistant affinity matrices.
	Use DIA acquisition to maximize target protein recovery, particularly for low-input, low-biotin samples.
**Optimize data analysis**	Avoid normalization between biotin-labeled samples and unlabeled controls.
	Consider combined MAR/MNAR imputation to retain conditionally downregulated proteins.
